# Analysis of meiotic segregation by triple-color fish on both total and motile sperm fractions in a t(1p;18) river buffalo bull

**DOI:** 10.1371/journal.pone.0232592

**Published:** 2020-05-04

**Authors:** Chiara Di Dio, Valentina Longobardi, Gianluigi Zullo, Pietro Parma, Alfredo Pauciullo, Angela Perucatti, James Higgins, Alessandra Iannuzzi

**Affiliations:** 1 Genetics and Genome Biology, University of Leicester, Leicester, United Kingdom; 2 Department of Veterinary Medicine and Animal Production, Federico II University, Naples, Italy; 3 Department of Agricultural and Environmental Sciences, Milan University, Milan, Italy; 4 Department of Agricultural, Forest and Food Sciences, University of Torino, Grugliasco (TO), Italy; 5 Laboratory of Animal Cytogenetics and Genomics, National Research Council (CNR)- ISPAAM, Naples, Italy; Sichuan Agricultural University at Chengdu, CHINA

## Abstract

Chromosomal aberrations are relatively frequent pathologies in both humans and animals. Among them, translocations present a specific meiotic segregation pattern able to give a higher percentage of unbalanced gametes that can induce fertility problems. In this study, the meiotic segregation patterns of 1p, 1q and 18 *Bubalus bubalis* chromosomes were analyzed in both total sperm fraction and motile sperm fraction of a t(1p;18) carrier and a control bulls by triple-color FISH analysis with a pool of specific BAC probes. The frequencies of each total sperm fraction products in the carrier resulting from alternate, adjacent I, adjacent II and 3:1 segregation were 39%, 20%, 1% and 38%, respectively. On the other hand, the frequencies of each motile sperm fraction products in the carrier resulting from alternate, adjacent I, adjacent II and 3:1 segregation were 93%, 5%, 0% and 2%, respectively. The frequencies of normal sperms in the carrier were 27% and 69% in total sperm fraction and motile sperm fraction, respectively. The frequencies detected in motile sperm fraction were also validated by comparison with bull’s progeny. To our knowledge, this is the first report on the meiotic segregation patterns in motile sperm fractions of *B*. *bubalis* bull carrying a chromosomal translocation. These data suggest that translocation has a very limited effect on aneuploidy in the gametes, and therefore, on the reproductive abilities of the bull.

## Introduction

The reproductive efficiency of bulls is considered one of the most important traits from an economic point of view. It gives an estimation of the amount of milk produced by a single dairy cow, influencing the frequency of conception [1]. After the insemination, the conception rate depends on physiologic and genetic factors, such as hormones, nutrition, level of energy and chromosomal aberrations (CA) [2,3]. The aneuploidies are often responsible of the embryonic and fetal death (genetically aberrant embryos formation), determining a lower calving rate in comparison to the fertilization rate [4]. Among these, the chromosomal aberrations (CA) are considered one of the major causes of embryonic loss in mammals and their incidence has been estimated around 7–10% in the embryos of domestic animals [5]. Those present in liveborn animals involve the sex chromosomes and balanced structural rearrangements as chromosomal translocations (CT), classified in Robertsonian or centric fusions (rob) and reciprocal translocations. CT involve the exchange of chromosomal material between the arms of heterologous chromosomes, thereby changing genetic positioning but, hardly ever, the amount of genetic material. They modify the meiotic division of germ cells giving race to normal and translocated (unbalanced spermatozoa) chromosomes formation by two type of segregation: 2:2 (alternate, adjacent I or adjacent II) and 3:1. This condition, in a carrier, can generate an elevated percentage of chromosomally unbalanced gametes that can reduce its reproductive success and increase the risk of transmitting chromosomal anomalies to offspring [6,7]. For this reason, since the discovery of the rob (1;29) in cattle [8] (5–10% reduction in fertility and responsible for approximately 3% of the unbalanced sperm and 4% of the unbalanced oocytes) [9,10], several countries have provided for cytogenetic monitoring of breeds, especially meat ones [11], detecting several chromosomal aberrations. Recent molecular studies suggest that rob(1;29) can also have a pericentric inversion or a transposition, in addition to the centric fusion [12,13].

Unlike cattle, autosomal CA, such as translocations, have rarely been reported in *Bubalus bubalis* (BBU) [14,15]. In Italian river buffalo, as often happens for other species also, there is no systematic karyotyping control for the bulls selected for progeny test. Therefore, the presence of undetected chromosomal aberrations in bulls, selected for Artificial Insemination (AI), may be spread in the population very quickly, with extremely negative results for the genetic progress of the species. Studies of CA in BBU bulls, selected for AI, have been much less frequent including a complex rearrangement involving BBU 1 chromosome fission followed by t(1p;23) (associated with embryonic death and reduced fertility) [16] and a t(1p;18) [17], object of this study. For this reason, the development of specific chromosome painting probes in BBU (river type) [18,19] is expected to improve cytogenetic screening in this species.

The purpose of this study was to compare the proportion of genetically unbalanced male gametes as well as the sperm aneuploidies of the translocated t(1p;18) BBU bull (2n = 50, XY) on both total sperm fraction (TSF) and motile sperm fraction (MSF), for the first time. Considering that, studies dealing with aneuploidies in bull spermatozoa have only been focused on the detection of sex chromosomes aneuploidies [20] or used as a tool for detection of the proportion of unbalanced spermatozoa involved in robs [21]. Triple color Fluorescent *in Situ* Hybridization (FISH) was used to study both the meiotic segregation patterns and the aneuploidy frequencies in BBU 1p, 1q and 18 chromosomes. For comparison, aneuploidy frequencies of the carrier and control bulls were evaluated in parallel on both TSF and MSF. Besides, the probability rate for progeny at birth was compared with the real progeny of the translocated bull (50 offsprings), as reported by Albarella et al. [17], demonstrating the importance of the mechanisms of meiotic segregation of translocations as a risk factor for pregnancy losses, birth defects and presence of cryptic carriers of chromosomal aberrations.

## Materials and methods

### Semen samples

Commercial cryopreserved semen straws, from two Italian Mediterranean river buffalo bulls, were obtained from the Italian Buffalo Breeders Association (ANASB), thus not requiring animal ethics committee approvals. The bulls examined in this study were previously karyotyped resulting in a t(1p;18) for the first one and a normal karyotype for the control.

### Swim-up technique and trypan blue test

Frozen spermatozoa were thawed at 37°C for 40 seconds and separated by the swim-up procedure in modified sperm thyroid's albumin lactate pyruvate medium [22] for 1 hour, to select only the motile population. Sperm motility and viability were evaluated immediately after thawing, while head decondensation was assessed after swim-up separation. Sperm motility was examined by phase-contrast microscopy (Nikon Diaphot 300) at 40 X magnification on a clean and dry glass slide overlaid with a coverslip and maintained on the thermo-regulated stage at 37°C. Any drifting of the specimen was permitted to stop and the percentage of motile spermatozoa was subjectively determined to the nearest 5% by analyzing four to five fields of view [23]. Sperm viability was assessed by the trypan blue/Giemsa technique as previously reported [24]. Briefly, 5 μl of semen and 5 μl of 0.27% trypan blue were spread on a clean slide fixed in 37% formaldehyde and stained with 7.5% Giemsa overnight. Sperm cells were microscopically evaluated at 40 X magnification (Nikon Diaphot 300). A total of 100 spermatozoa were analyzed per slide and the percentage of alive sperm with an intact acrosome was recorded.

### Slide preparation and decondensation

Preparation and decondensation of spermatozoa were conducted according to the protocol described by Pauciullo et al. [25] with slight modifications. Briefly, the semen of each animal, obtained from both entire samples and samples undergoing swim-up technique, was washed three times in an equal volume of PBS (pH 7.4) containing 6 mmol l^−1^ EDTA and fixed in 3:1 methanol: acetic acid. A 15μl droplet of the fixed suspension was placed on a clean microscopic slide and air-dried at room temperature for 3 hours. In this way, four different kinds of samples were prepared: 1) TSF from the carrier of t(1p;18); 2) MSF from the carrier of t(1p;18); 3) TSF from the control; 4) MSF from the control. Sperm DNA was denatured by immersion in 3 mmol l^−1^ NaOH at room temperature and, the optimal decondensation time was determined experimentally resulting in 3 min for slides 1 and 3, whereas 2 min for slide 2 and 4, respectively. After decondensation, all the slides were washed in distilled water, dehydrated by a series of ethanol solutions (70, 80, and 96%) at −20°C for 2 min each, and air-dried before hybridization.

### Probes preparation, hybridization and signal analysis

**Fig 1:**
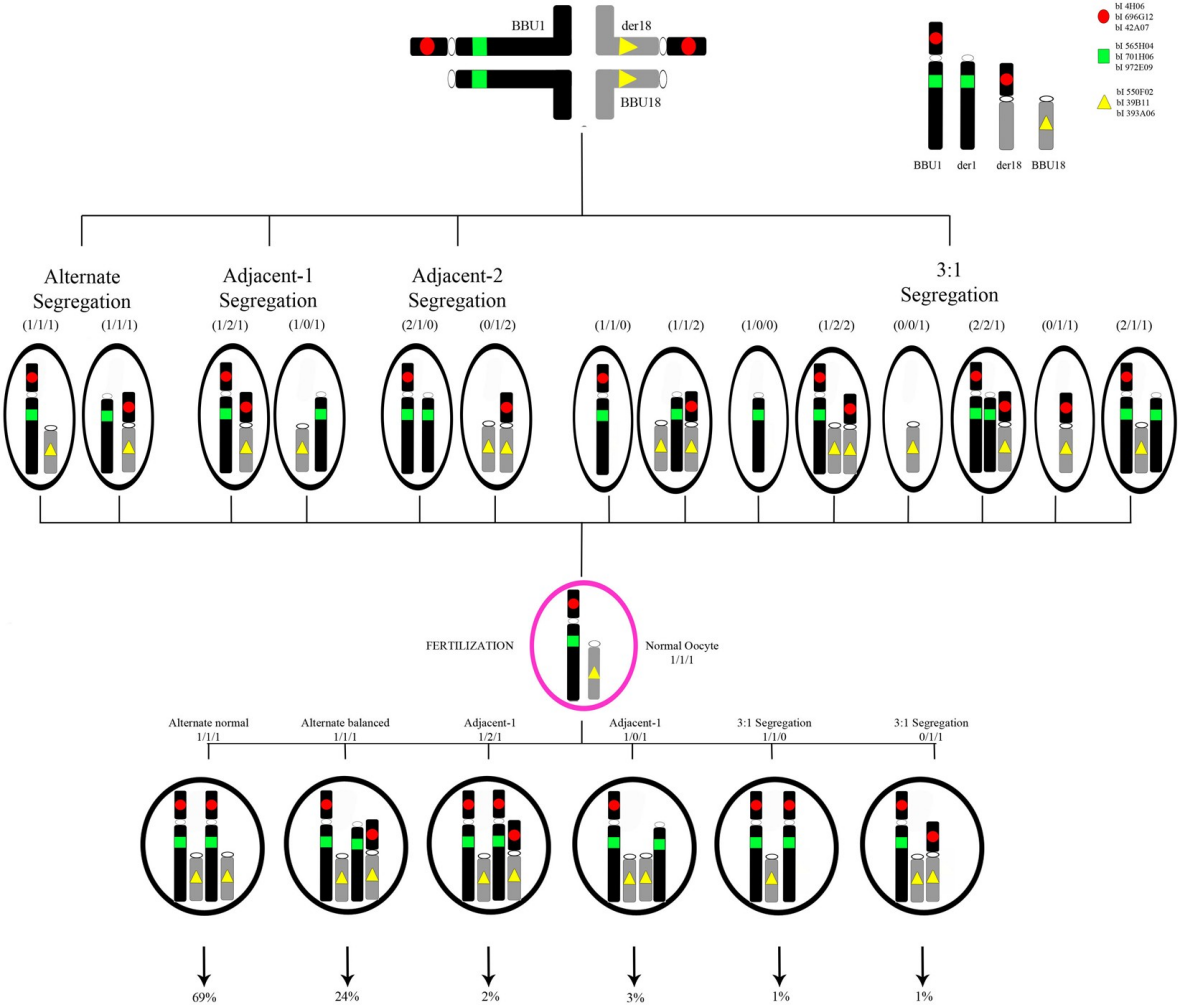
**Illustration of the various gametes produced by 2:2 and 3:1 segregation mechanisms of *Bubalus bubalis* (BBU) 1 and 18 chromosomes involved in the translocation, with localization of the DNA probes used in the meiotic segregation assay (upper pane): Red circle (R) for the 1**^**st**^ **pool, green square (G) for the 2**^**nd**^ **pool and yellow triangle (Y) for the 3**^**rd**^ **pool.** The signal pattern of FISH and the corresponding fluorescent phenotypes are also indicated, while interstitial crossing-overs were not considered. Corresponding schematic representation of the fertilization between a normal river buffalo oocyte and t(1p;18) carrier sperms detected by FISH analysis for MSF, where for each type of embryo is indicated the success rate (%) according to our results.

**Fig 2:**
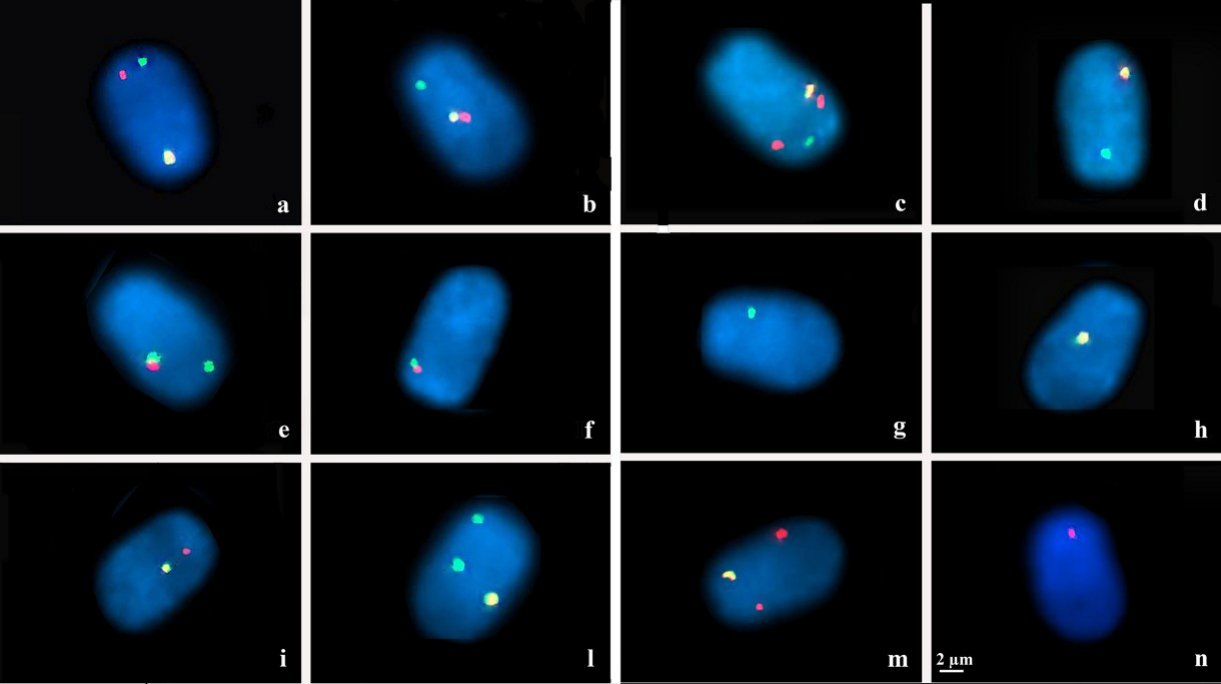
**Representative sperm nuclei for bull *Bubalus bubalis* t(1p;18) carrier with 1**^**st**^ **(BBU 1p), 2**^**nd**^ **(BBU 1q) and 3**^**rd**^ **(BBU 18q) pool stained in (red) R, (green) G and yellow (Y), respectively. a** Normal sperm nucleus with 1/1/1 fluorescent phenotype and separate signals. **b** Balanced sperm nucleus with 1/1/1 fluorescent phenotype and R and Y signals close to each other. **c** Adjacent I sperm nucleus with 1/2/1 fluorescent phenotype. **d** Adjacent I sperm nucleus with 1/0/1 fluorescent phenotype. **e** Adjacent II sperm nucleus with 2/1/0 fluorescent phenotype. **f** 3:1 sperm nucleus with 1/1/0 fluorescent phenotype. **g** 3:1 sperm nucleus with 1/0/0 fluorescent phenotype. **h** 3:1 sperm nucleus with 0/0/1 fluorescent phenotype. **i** 3:1 sperm nucleus with 0/1/1 fluorescent phenotype. **l** Other sperm nucleus with 2/0/1 fluorescent phenotype. **m** Other sperm nucleus with 2/0/1 fluorescent phenotype. **n** Other sperm nucleus with 0/2/1 fluorescent phenotype.

Each probe used in the study consisted of a pool of three Bacterial Artificial Chromosome (BACs) selected in a contig (maximal distance between clones: 600 Kb), to increase the size of the sequence covered by the probe (around 1 Mb) and thus the intensity of the FISH signals (Table 1). They were obtained from the INRA bovine BAC library [26] and their specificity was first tested on both bovine and river buffalo lymphocyte banded metaphases (S1 Fig). The schematic representation of the translocation and localization of the BACs, used in this study, is illustrated in Fig 1. DNA isolation was performed using CHORI (Children’s Hospital Oakland Research Institute) recommended protocol. Preparation of probes and hybridization by Triple-colour FISH analysis were carried out according to the protocols described by Iannuzzi et al. [27,28] with slight modifications. Briefly, the probes were labeled with Biotin-16-dUTP and Digoxigenin-11-dUTP by Bio-Prime Array kit (Invitrogen) as showed in Table 1. Each probe was denatured for 10 min at 70°C and then pre-annealed by incubation at 37°C for 60 min. For each slide, three probes were hybridized simultaneously on decondensed sperm heads, covered with 22-mm coverslips, sealed and incubated for 24 h at 37°C in a moist chamber. Post-hybridization washes involved two series of 5 min incubations in 50% formamide in 2X SSC at 45°C followed by two series of 5-min incubations in 2XSSC at 45°C. Hybridization sites of labeled probes were visualized by indirect staining using FITC-avidin and TRIC-anti-digoxigenin antibody, incubating the slide in a dark moisture chamber at 37°C for 1h. Finally, spermatozoa were counterstained with Vectashield DAPI H 1000 (Vector Lab) antifade solution. The slides were analyzed under a Leica DM 5500 (Leica Microsystems) fluorescence microscope equipped with 100 X lens, triple bandpass specific filters (DAPI, FITC, Texas Red) and high-sensitivity monochrome camera. Digital images were captured in grey-scale and false colors were created by Cytovision-Leica specific software, giving different types of gametes (Fig 2).

**Table 1:** BAC probes with their genome view (according to NCBI) used for triple colour FISH analysis.

PROBE	TOTAL LENGHT (Kb)	*Bos taurus* UMD 3.1.1 Genome localization (bp)	CHROMOSOMAL LOCALIZATION	LABEL	IMPOSED COLOUR
**1**^**st**^ **pool**					
bI 0004H06	83,78	22,452,603–22,536,386	*BTA* 27q 13		
bI 0696G12	122,39	23,090,214–23,212,604	*BBU* 1p 13	Digoxigenin	Red
bI 0042A07	242,34	23,287,629–23,529,972			
**2**^**nd**^ **pool**					
bI 0565H04	110,73	284,010–394,741	*BTA* 1q 1.3–1.4		
bI 0701H06	139,82	393,355–533,183	*BBU* 1q 1.3–1.4	Biotin	Green
bI 0972E09	115,29	43,977–159,269			
**3**^**rd**^ **pool**					
bI 0550F02	152,78	45,041,967–45,194,755	*BTA* 18q 1.5–2.1	Biotin	
bI 0039B11	100,79	44,410,860–44,511,655	*BBU* 18q 1.5–2.1	+	Yellow
bI 0393A06	147,11	43,921,537–44,068,649		Digoxigenin	

### Statistical analysis

Chi-square Test [29] was used to compare: 1) the frequencies between sperm selection methods within each bull; 2) the frequencies between carrier and control bull within each sperm selection methods; 3) the carrier alternate segregation sperm frequencies (normal and balanced sperms) and bull’s progeny. The *P*-value of less than 0.01 was considered significant.

## Results

### Trypan blue test

The semen analysis data in both bulls (carrier and control) was similar for the sperm motility (60.0±0.0) as well as for the sperm viability (65.0±0.0). However, the translocated bull showed a great percentage (35%) of bent tail abnormalities on live sperm.

### FISH analysis

Fig 1 shows the predicted segregation patterns of BBU chromosomes 1 and 18. The corresponding segregation analysis, illustrated in Table 2, was achieved on an average of 10,000 spermatozoa (evaluated in the step of 2,000 and 5,000 also) for TSF and MSF of the carrier and 2,000 for TSF and MSF of the control samples. The efficiency of hybridization was about 99.7% where only well-decondensed sperm heads, exhibiting high-intensity signals, were considered for the analysis. The meiotic segregation pattern for the t(1p;18) carrier was determined by the presence of triple color FISH signals that corresponded to chromosomes 1p (red signal), 1q (green signal) and 18 (yellow signal), as shown in Fig 2. In order to distinguish alternate normal (1 and 18 BBU chromosomes) from balanced (der1 and der18) sperms, we have considered as normal sperms all those who presented three detached signals (Fig 2A) and, balanced sperms all those presented yellow and red signal close to each other and green signal detached (Fig 2B). We have evaluated them also in the control bull to validate our hypothesis. In this way, for the carrier, the frequencies of alternate, adjacent I, adjacent II and 3:1 segregation were 39%, 20%, 1% and 38%, for TSF, and 93%, 5%, 0% and 2% for MSF, respectively. In the control, they were 98%, 0%, 0% and 2% for TSF and 98%, 1%, 0% and 1% for MSF (Table 2). For this reason, we have shown that 27% of TSF of the carrier has resulted normal (in comparison to the control with 97%), while the 12% were balanced (in comparison to the control with 1%). On the other hand, 69% of the MSF of the carrier was normal (in comparison to the control with 97%), while the 24% were balanced (in comparison to the control with 1%). However, in TSF of the carrier, we have found a high percentage of Adjacent I (20%) and 3:1 (38%) segregations not detected in MSF. Other possible combinations were observed only in the TSF of the carrier (2%).

**Table 2:** Sperms resulting from meiotic segregation on both t(1p;18) and control bulls, detected on total and motile sperm fractions.

PATTERNS OF SEGREGATION	FLUORESCENT PHENOTYPES	ASSOCIATED GENOTYPE	No. of TOTAL SPERM FRACTIONS t(1p;18)	%	No. of TOTAL SPERM FRACTIONS (ctr)	%	No. of MOTILE SPERM FRACTIONS t(1p;18)	%	No. of MOTILE SPERM FRACTIONS (ctr)	%
Alternate	(1/1/1)	Normal n = 25, +BBU1; +BBU18; -der1; -der18	2,681	27	2,264	97	6,906	69	2,070	97
	(1/1/1)	Balanced n = 25; +der1; +der18; -BBU1; -BBU18	1,223	12	16	1	2,443	24	21	1
		Alternate TOT	3,904	39	2,280	98	9,349	93	2,091	98
Adjacent I	(1/2/1)	n = 25; +BBU1; +der18; -BBU18; -der 1	841	8	0	0	155	2	27	0
	(1/0/1)	n = 25; +BBU18; +der1; -BBU1; -der18	1,131	11	10	0	308	3	2	0
		Adjacent I TOT	1,972	20	10	0	463	5	29	1
Adjacent II	(2/1/0)	n = 25; +BBU1; +der1; -BBU18; -der18	147	1	0	0	8	0	0	0
	(0/1/2)	n = 25; +BBU18; +der18; -BBU1; -der1	0	0	0	0	0	0	0	0
		Adjacent II TOT	147	1	0	0	8	0		0
3:1 Segregation	(1/1/0)	n = 24; +BBU1; -BBU18; -der1; -der18	1,556	15	18	1	71	1	7	0
	(1/0/0)	n = 24; +der1; -BBU1; -BBU18; -der18	506	5	0	0	10	0	0	0
	(0/1/1)	n = 24; +der18; -BBU1; -BBU18; -der18	1,241	12	0	0	108	1	0	0
	(0/0/1)	n = 24; +BBU18; -BBU1; -der18; -der1	504	5	22	1	18	0	8	0
		3:1 Segregation TOT	3,807	38	40	2	207	2	15	1
Other	(2/0/1)	n = 25; +BBU18; +der1; -BBU1; -BBU18	7	0	0	0	0	0	0	0
	(0/2/1)	n = 25; +der18; +der1p; -BBU1; -BBU18; -der1	131	1	0	0	0	0	0	0
	(0/1/0)	n = 24; +der1p; -BBU1; -der1; -BBU18; -der1	78	1	0	0	0	0	0	0
		Other TOT	216	2	0	0	0	0	0	0
** **	** **	**TOT**	**10,046**	** **	**2,330**	** **	**10,027**	** **	**2,135**	** **

### Statistical analysis

**Fig 3:**
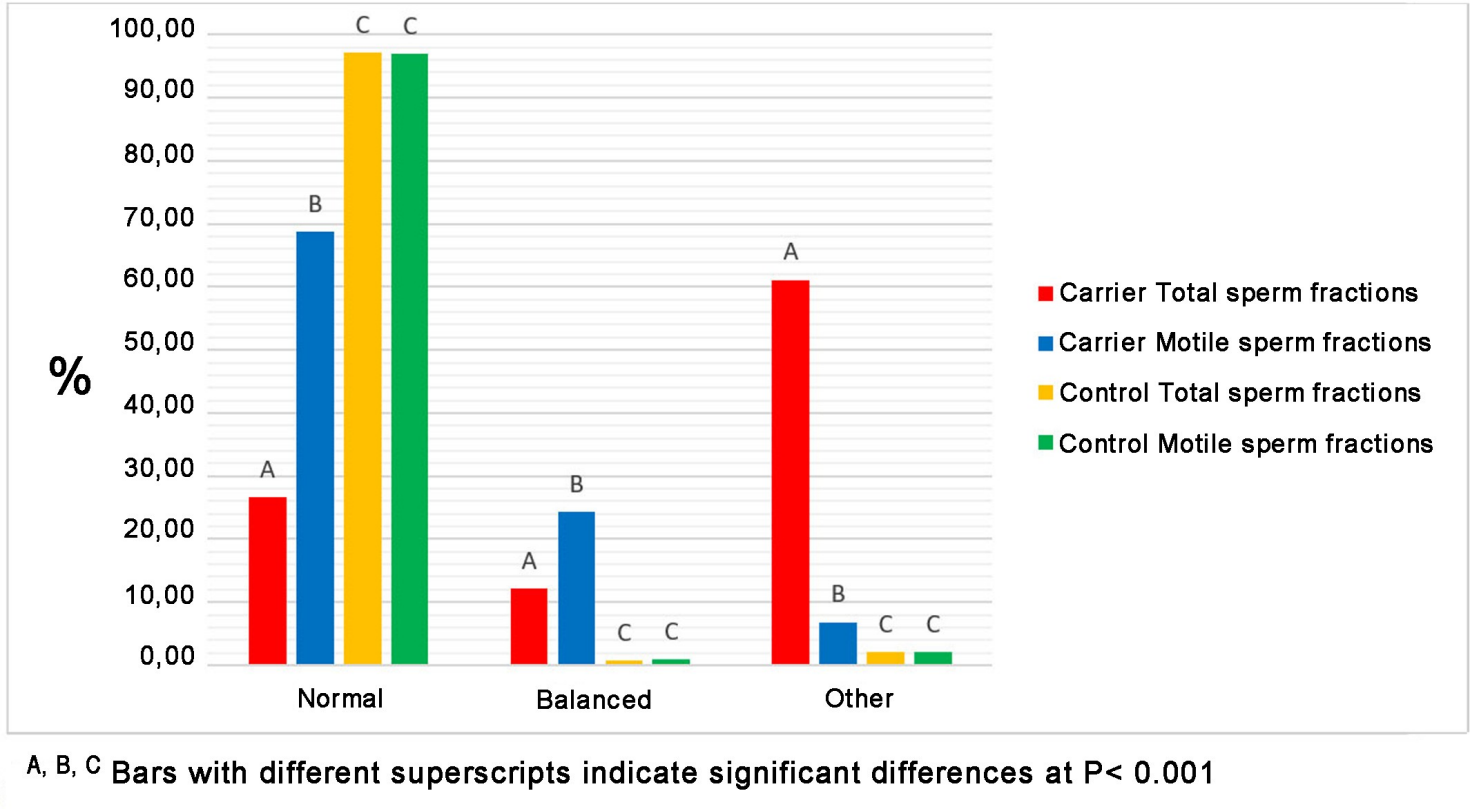
Differences between both sperm selection methods and bulls by Chi-square Test.

Significant differences (P <0.001) were observed both between sperm selection methods and between bulls (Fig 3). No significant differences were observed comparing Alternate segregations (normal and balanced sperms) of the carrier and its offspring reported by Albarella et al. [17] (Table 3).

**Table 3:** Alternate carrier sperms segregation and bull’s progeny differences by Chi-square Test.

Alternate segregations	Total sperm fractions n (%)	Motile sperm fractions n (%)	Bull’s progeny* n (%)
**Normal**	2,681 (69)^A^	6,906 (74)^B^	35 (70.0)^A^^B^
**Balanced**	1,223 (31)^A^	2,443 (26)^B^	15 (30.0)^A^^B^
**Total**	3,904	9,349	50

^A,B^ Values with different superscripts within rows are different P < 0.001

* Data reported by Albarella et al., 2013

## Discussion

The analyses performed in this study aimed to define the quality of viable sperms derived from a buffalo bull with a chromosomal translocation compared to a control bull. No significant differences were recorded in sperm motility and viability between the carrier and control bulls. However, the carrier bull showed a higher proportion of bent tails. In swamp buffalo (*Bubalus bubalis* 2n = 48), it is known that sperm abnormalities, in bulls selected for AI, should not exceed 15% of tail defects [30,31] whereas, to our knowledge, no information is available for the river type. Nevertheless, sperm defects involving tail are mostly filtered out in the female reproductive tract reaching the oviduct, reducing competition with normal cells for fertilization [32]. In a recent study, the relationship between sperm quality and *in vivo* fertility was examined on the Italian Mediterranean buffalo bull. The results of this study showed that bent tails may be associated with sperm accumulation in the ampullae, resulting in sperm aging which can decrease sperm DNA quality, hence fertility [33]. Regardless of the number of normal sperms present, fertility decreases with >30% morphologically abnormal sperm or >20% head defects [34].

Cytological studies on chromatin architecture often utilize 3D FISH analysis to conserve the shape and three-dimensional structure of the nuclei [35], considering the cell nucleus has a compartmentalized structure assembled by Chromosome Territories and Inter-Chromatin Compartment. Chromatin architecture may be also studied with 2D FISH analysis proving that 1) individual chromosomes occupy distinct territories [36]; 2) each chromosome has a defined intra-nuclear localization and the relative positioning of chromosomes is not-random [37]. For this reason, we have chosen 2D FISH analysis combined with a pool of BACs, to obtain more precise and distinct spot signals not revealing extended, string-like structures or loops, to evaluate sperm segregations in the best way. We have validated our hypothesis to consider as normal sperms (normal chromosomal structure) all those presented three detached signals (Fig 2A) and balanced sperms (abnormal chromosomal structure) all those presented yellow and red signal close to each other and green signal detached (Fig 2B). Furthermore, we have evaluated them in the control bull also, which possessed 97% normal sperms and the 1% of balanced sperms (most probably induced by a coincidence due to the sperm rotation) in both sperm selected methods. For this reason, the carrier possessed a significantly higher percentage of balanced sperms on both sperm selected methods in comparison to the control one. If there had been alterations of the signals due to the 2D FISH analysis (overlapping signals), we should have had 2 yellow signals for the normal sperms and 1 green and 1 yellow signals for the balanced sperms; but we have never detected these combinations in all sperms counted, as shown in Fig 2.

We counted 10,000 sperms of the carrier, for each sperm selected methods, to have more precise data due to the various segregation patterns detected (Table 2), in comparison to the control where 2,000 sperms (98% Alternate) were sufficient for a good evaluation. We have evaluated carrier segregations at 2,000 and 5,000 total sperms also, finding no change in the percentage of sperm segregation patterns detected in 10,000 on both selected methods. In this way, we analyzed the frequencies of numerical chromosomal aberrations derived from a translocation in both total and swim-up sperm fractions, mainly to examine the efficiency of swim-up in the elimination of sperms with numerical chromosomal aberrations, comparing also the results with the offspring according to the results reported in Albarella et al. [17] (Table 3).

The results of this study demonstrated that the swim-up separation was effective in reducing the percentage of chromosomal aberration in the carrier bull compared with the control one, improving sperm quality (Fig 3). This result was also validated by significant differences found between TSF and MSF of both Adjacents I and 3:1 Segregation of the carrier (Table 2). TSF of the carrier showed 20% of Adjacent I and the 38% of 3:1 Segregation, most probably due to the presence of massive unbalanced non-motile sperms, not detected in MSF. On the other hand, no significance has been found between TSF and MSF of the control bull, where about 98% of sperms have resulted with normal chromosome constitution (Table 2) in both fractions, while 2% of sperms resulted in aneuploidy for BBU 1 and 18, giving a frequency of autosomal disomies approximately 0.04% per chromosome. This data showed the frequencies of autosomal aneuploidies are generally low and comparable with the results showed by Rybar detected in 6 normal bulls [38], while they resulted lower in comparison with standard findings in humans [39,40] and pigs [41].

Finally, our results are in accordance with an earlier study in humans in which swim up reduced the proportion of sperm with chromosomal aberrations and the rate of sperm disomies and diploids [42]. Furthermore, a correlation between the percentage of MSF (Alternate segregation) of the carrier and its offspring has been demonstrated (Table 3). In this case, 74% of sperms had a normal chromosome constitution, while 26% had the same translocation as the carrier (balanced chromosome constitution), in line with data of bull’s progeny (70% normal and 30% translocated) reported by Albarella et al. [43], as shown in Table 3.

## Conclusions

Different biotechnologies as swim-up techniques, FISH analysis, a pool of BAC probes have been used together to get a more precise and complete segregation analysis. To our knowledge, this is the first report on the meiotic segregation patterns of a BBU bull carrying a CT that shows the strict connection among the MSF selected by the swim-up method, the chromosome constitution and the progeny. These data suggest that translocation may have a very limited effect on aneuploidy in the gametes, and therefore, on the reproductive abilities of the bull. Finally, thanks to the cytogenetic monitoring of BBU bulls, CA can be easily detected, preserving the genetic selection of reproducers.

Further studies are needed to increase the dataset, to better understand the correlation between motility and chromatin integrity.

## Supporting information

S1 Fig **FISH analysis on *Bubalus bubalis* RBH (R-banded Hoechst) metaphase spreads using the three pools of BAC probes: Red signals represent the 1**^**st**^ **pool located on BTA 27, green signals represent the 2**^**nd**^ **pool located on BTA 1 and yellow signals represent the 3**^**th**^ **pool located on BTA 18.** The arrows represent the position of centromere in each chromosome.
(TIF)
