# Supplementary figures and images for: Analysis of meiotic segregation by triple-color fish on both total and motile sperm fractions in a t(1p;18) river buffalo bull
Source: PLoS One. 2020 May 4;15(5):e0232592. doi: 10.1371/journal.pone.0232592 (PMC7197801; doi:10.1371/journal.pone.0232592)

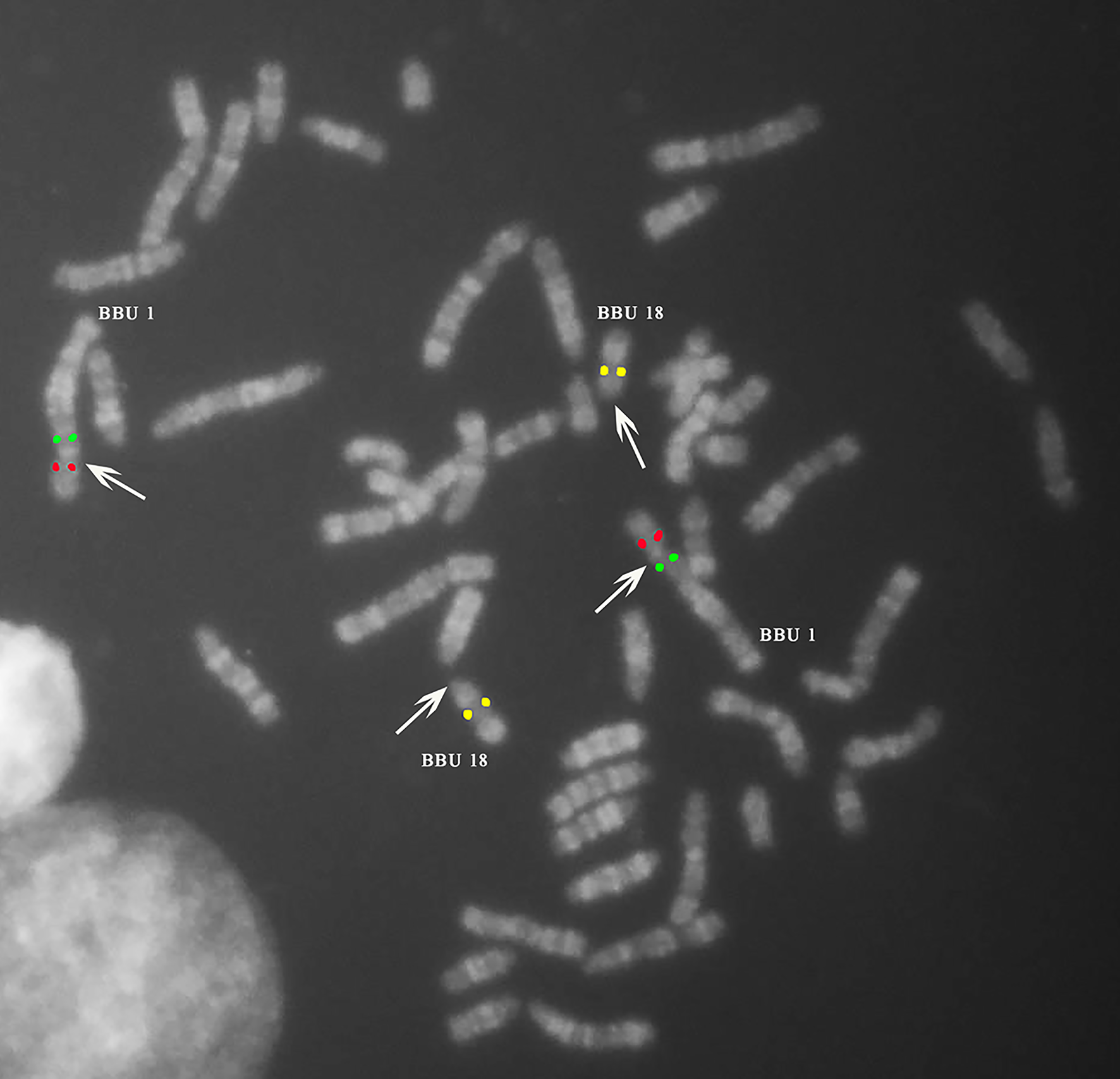

Supplement: S1 Fig — FISH analysis on Bubalus bubalis RBH (R-banded Hoechst) metaphase spreads using the three pools of BAC probes: Red signals represent the 1st pool located on BTA 27, green signals represent the 2nd pool located on BTA 1 and yellow signals represent the 3th pool located on BTA 18. The arrows represent the position of centromere in each chromosome. (TIF) [file pone.0232592.s001.tif]
